# MTHFR C677T and Brain MRI Patterns Across Cognitive and Vascular Disorders: A Systematic Review

**DOI:** 10.3390/biomedicines14102368

**Published:** 2026-10-17

**Authors:** Andreea Alexandra Gogu, Any Axelerad, Anca Elena Gogu, Marius Benta, Oana Neda-Stepan, Ana Cristina Bredicean

**Affiliations:** 1 Doctoral School, University of Medicine and Pharmacy “Victor Babeș”, 300041 Timișoara, Romania; andreea.gogu@umft.ro; 2 First Department of Neurology, “Pius Brînzeu” Clinical Emergency County Hospital, 300723 Timișoara, Romania; 3 Department of Neurology, General Medicine Faculty, “Ovidius” University, 900470 Constanța, Romania; docuaxi@yahoo.com; 4 Department of Neurosciences, First Division of Neurology, University of Medicine and Pharmacy “Victor Babeș”, 300041 Timișoara, Romania; 5 Department of Radiology, “Pius Brînzeu” Clinical Emergency County Hospital, 300723 Timișoara, Romania; marius.benta@umft.ro; 6 Department of Neurosciences, Division of Psychiatry, University of Medicine and Pharmacy “Victor Babeș”, 300041 Timișoara, Romania; oana.neda-stepan@umft.ro (O.N.-S.); bredicean.ana@umft.ro (A.C.B.)

**Keywords:** MTHFR C677T polymorphism, methylenetetrahydrofolate reductase (NADPH2), Alzheimer’s disease, mild cognitive impairment, dementia, vascular, cerebral small vessel diseases, magnetic resonance imaging, homocysteine, white matter, brain atrophy, meta-analysis as topic

## Abstract

**Background:** Elevated plasma homocysteine, arising from impaired one-carbon metabolism, has been implicated in cerebral microvascular injury and in neurodegeneration. The methylenetetrahydrofolate reductase (MTHFR) C677T polymorphism is the commonest genetic determinant of homocysteine concentration, but whether it leaves a measurable structural footprint on the brain remains unresolved. We synthesised genotype-stratified structural MRI findings in Alzheimer’s disease, mild cognitive impairment, and vascular cognitive disorders. **Methods:** Four sources (PubMed, Europe PMC, OpenAlex, Semantic Scholar) were searched up to 20 September 2026. Eligible reports gave C677T-stratified structural MRI outcomes in adults with clinically defined cognitive disorders. Quality was appraised with an adapted Newcastle–Ottawa Scale, and exploratory random-effects meta-analyses supplemented a narrative synthesis. **Results:** Thirteen studies (3880 participants across partly overlapping cohorts) were included. Moderate-to-severe white matter lesions were modestly more frequent in TT homozygotes, consistently across contributing studies, with no consistent excess in heterozygotes. Regional volume findings were inconsistent and largely confined to one cohort, whereas whole-brain atrophy rate, hippocampal and medial temporal measures, lacunes, microbleeds, and diffusion metrics showed no genotype-only association. Homocysteine was higher in T-allele carriers wherever measured. **Conclusions:** The evidence is more compatible with a modest association with vascular white matter injury than with a reproducible neurodegenerative imaging phenotype and does not support genotype-guided clinical decision-making. Longitudinal, biomarker-characterised cohorts with harmonised imaging, adequately powered genotype groups, and measured nutritional status are the main priorities.

## 1. Introduction

Cognitive impairment in later life is a leading cause of dependency in older adults, and its two commonest substrates—Alzheimer’s disease (AD) and cerebrovascular disease—rarely occur in isolation: neuropathological series show that mixed Alzheimer-type and vascular pathology is the rule rather than the exception in late life [1]. Vascular cognitive impairment and dementia form a spectrum in which cerebral small vessel disease (CSVD) is the dominant substrate [2,3]. Precisely because the two processes coexist, a factor acting preferentially on one of them is difficult to identify from clinical phenotype alone, and tissue-level markers are needed. Structural magnetic resonance imaging (MRI) supplies them: hippocampal, medial temporal, and posterior cortical atrophy index Alzheimer-type neurodegeneration, whereas white matter hyperintensities (WMHs), lacunes, and cerebral microbleeds are the standardised neuroimaging signatures of CSVD [4,5]. WMH burden independently predicts incident cognitive impairment and dementia in prospective cohorts [6,7]. A risk factor that accelerates either pathway should therefore be detectable as genotype-dependent differences in these imaging phenotypes, in parallel with or before clinical decline.

One modifiable candidate pathway is one-carbon metabolism. The 5,10-methylenetetrahydrofolate reductase gene (MTHFR) encodes the enzyme that reduces 5,10-methylenetetrahydrofolate to 5-methyltetrahydrofolate, the methyl donor required for the remethylation of homocysteine to methionine. The common C-to-T transition at nucleotide 677 (rs1801133; Ala222Val) produces a thermolabile enzyme with markedly reduced activity in homozygotes and was first described as a candidate genetic risk factor for vascular disease three decades ago [8]. Carriers of the T allele, and particularly TT homozygotes, display higher plasma total homocysteine concentrations, an effect that is amplified when folate or vitamin B12 status is marginal [8,9]. Homocysteine exerts excitotoxic, pro-oxidant, and pro-thrombotic effects and injures the cerebral microvasculature, and higher concentrations have been associated with incident AD in prospective cohorts [10], with both Alzheimer-type and cerebrovascular pathology at autopsy [11], and with regional brain volume deficits in older adults [12]; an international consensus statement regards the evidence linking homocysteine to cognitive decline as sufficient to justify intervention research [13]. The variant therefore plausibly connects to both neurodegenerative and vascular pathways, with nutritional status as a modifier of its biochemical expression.

Current knowledge on MTHFR and dementia derives mainly from case–control genetic association studies. Meta-analyses report that the 677T allele modestly increases the risk of AD and of vascular dementia (VaD), with pooled odds ratios of roughly 1.1 to 1.4 across genetic models and with associations concentrated in Asian populations, late-onset AD, and apolipoprotein E (APOE) ε4 carriers rather than in European populations [14,15,16,17]. No association has been demonstrated with mild cognitive impairment (MCI) under any genetic model [18], and MTHFR appears among the candidate loci in syntheses of genetic susceptibility to VaD in Asian populations [19]. These estimates remain contested: several large European cohorts found no link between C677T and cognitive decline, between-study heterogeneity is substantial, and publication bias and population stratification are unresolved concerns. More fundamentally, susceptibility odds ratios describe who enters a diagnostic category; they do not identify the tissue-level process involved, nor do they indicate whether patients who already have a cognitive disorder show different patterns or rates of structural brain change according to genotype. Genotype-stratified imaging addresses that question directly because it compares brain structure between genotypes within a clinically defined population and can therefore separate a vascular from a neurodegenerative locus of effect—a distinction that disease-susceptibility designs cannot make.

Two lines of evidence suggest that the variant could leave a structural footprint. First, higher plasma homocysteine has been mapped to regional brain volume deficits in older adults, most clearly in people with MCI [12], and to more severe medial temporal and cerebrovascular pathology at autopsy [11]. Second, randomised trials have shown that homocysteine-lowering with high-dose B vitamins slows whole-brain atrophy in MCI [20] and reduces grey matter atrophy in AD-vulnerable regions in participants with elevated baseline homocysteine [21], supporting investigation of a modifiable nutritional pathway without establishing genotype-specific treatment efficacy; pooled trial data show no overall effect of homocysteine lowering on cognitive test scores, which makes the identification of susceptible subgroups more rather than less important [22]. Genotype-stratified imaging studies can complement these trials, although population structure, selection, and alternative biological pathways complicate causal interpretation. Contemporary biological definitions of AD emphasise amyloid and tau biomarkers over clinical syndromes [23,24], making it relevant to determine whether a homocysteine-related variant modulates neurodegeneration independently of, or through, vascular co-pathology.

Despite this rationale, the imaging genetics literature on MTHFR has not been addressed by the existing disease-susceptibility syntheses. Meta-analyses of C677T in relation to dementia risk [14,15,16,17,18,19], to CSVD presence [25], and to lacunar infarction [26] exist, but none has pooled or narratively synthesised genotype-stratified MRI outcomes in cognitively impaired populations. Primary studies are scattered across neuroimaging, genetics, geriatric, and cerebrovascular journals, use disparate outcome measures ranging from voxel-wise tensor-based morphometry to visual white matter rating scales, and have been conducted in populations that differ in ancestry, folate fortification status, and diagnostic criteria. Individual reports have reached seemingly discordant conclusions, with some describing genotype-dependent white matter volume loss and others reporting no association with WMHs. A systematic review conducted according to PRISMA 2020 standards [27] is needed to summarise the evidence, make eligibility and appraisal transparent, pool comparable data, and identify methodological gaps.

This systematic review aimed to identify, appraise, and synthesise accessible primary studies examining the association between the MTHFR C677T polymorphism (alone or together with A1298C) and structural brain MRI alterations in adults with AD, MCI, or vascular cognitive impairment, including symptomatic CSVD cohorts that represent the substrate of VaD [2,28]. Specifically, we asked whether carriers of the 677T allele differ from CC homozygotes in grey matter, hippocampal or cortical volume, white matter integrity, or CSVD lesion burden on MRI and whether the pattern of any such difference distinguishes neurodegenerative from vascular phenotypes. Diagnostic categories were accepted as defined by the primary studies, most of which used established criteria for MCI due to AD [29], probable AD [30], or neuroimaging-based CSVD [4]. Secondary aims were to pool comparable genotype-stratified data where feasible and to establish whether studies examined homocysteine as a mediator and APOE ε4 as an effect modifier. The novelty of the review lies in this focus on imaging phenotypes within already affected populations, an evidence base that the existing susceptibility meta-analyses [14,15,16,17,18,19,25,26] do not cover.

## 2. Materials and Methods

### 2.1. Review Design and Reporting

This review combined three components that address different aspects of a single question, and the relationship between them determines how the findings should be read. A narrative synthesis, structured by clinical population and by structural outcome domain, carries the primary analysis because the included studies used heterogeneous imaging measures, genetic models, and covariate sets that cannot be reduced to one effect size. A structured quality appraisal was applied to every included report, so the weight attached to an individual finding reflects design, genotype group size, covariate adjustment, and completeness of reporting rather than statistical significance alone. An exploratory meta-analysis was then restricted to the two outcomes for which at least three studies reported comparable unadjusted, genotype-stratified data; its purpose is to summarise the direction and consistency of those particular counts, not to produce a confirmatory pooled effect. The three components are hierarchical rather than parallel: the narrative synthesis frames the evidence, the appraisal qualifies it, and the pooled estimates describe the one domain in which quantitative summary was feasible. Reporting followed the PRISMA 2020 statement [27] and, for the narrative components, the Synthesis Without Meta-analysis (SWiM) guideline [31]; the data items relating to genotyping and genetic models were informed by the STREGA reporting recommendations for genetic association studies [32].

A written protocol specifying the review question, eligibility criteria, search sources, data items, quality appraisal, and synthesis plan was prepared before the searches were run; the protocol was registered with OSF, with the registration code https://osf.io/3ct69 (accessed on 14 October 2026). Ethical approval was not required because the review used published aggregate data.

### 2.2. Eligibility Criteria

Eligible designs were primary observational human studies (cross-sectional, case–control, or cohort) and secondary analyses of prospective cohorts or randomised trials in which a genotype-stratified imaging analysis was reported. Participants were adults with AD dementia, MCI or amnestic MCI, VaD or vascular cognitive impairment, or symptomatic CSVD in which cognitive dysfunction was part of the clinical presentation or was measured. Mixed cohorts of cognitively normal, MCI, and dementia participants were eligible when results were adjusted for or stratified by diagnosis. Accepted diagnoses included established AD and MCI criteria [29,30] and vascular cognitive impairment harmonisation standards [28]. Symptomatic CSVD without quantified cognition was retained when cognitive dysfunction was an explicit recruitment manifestation but was treated as less direct evidence for cognitive impairment. Imaging-defined leukoaraiosis, stroke, or population-based cohorts without an eligible cognitive population were excluded.

Reports had to describe C677T genotyping and at least one structural MRI outcome by genotype or allele dose. Outcomes included regional or whole-brain volume, cortical morphometry, hippocampal or medial temporal measures, white matter hyperintensities or lesions, lacunes, microbleeds, and diffusion measures. Visual lesion scales, including Fazekas ratings [33], were eligible. A1298C and other variants were recorded when reported alongside C677T. Purely biochemical, cognitive, cerebrospinal fluid, functional imaging, or gene expression findings without an eligible genotype–structural MRI contrast were excluded, as were polygenic or Mendelian randomisation analyses that did not report a C677T-specific imaging contrast. Reviews, conference abstracts, case reports, uncontrolled case series, narrative perspectives, and preclinical studies were ineligible. No language or geographic restrictions were applied.

### 2.3. Information Sources and Search Strategy

Four bibliographic sources were searched from inception to 20 September 2026: PubMed (MEDLINE), Europe PMC (including full-text indexing of open-access articles and preprints), OpenAlex, and Semantic Scholar. Each search combined a gene block (MTHFR, methylenetetrahydrofolate reductase, C677T, rs1801133, A1298C, rs1801131) with a clinical block (dementia, Alzheimer, cognitive impairment or decline, vascular cognitive impairment, small vessel disease, leukoaraiosis, white matter lesions or hyperintensities) and an imaging block (MRI, magnetic resonance, neuroimaging, atrophy, grey or white matter, hippocampal measures, cortical thickness, brain volume, diffusion tensor imaging, morphometry, microbleeds, lacunes). The PubMed strategy was deliberately sensitive and required the gene block together with either the clinical or the imaging block, whereas the Europe PMC, OpenAlex, and Semantic Scholar strategies required all three blocks. All result pages were downloaded in full without relevance-ranked truncation. Forward citation searching of every included report was performed in OpenAlex (959 citing records screened by title), and the reference lists of included reports and of related meta-analyses [17,25,26] were checked. The full query strings, dates, record counts, and the record-level decision ledger are available from the corresponding author upon reasonable request. Embase, Web of Science, Scopus, trial registers, and Chinese-language databases (CNKI, Wanfang, SinoMed) were not searched, and this is the principal limitation of the search strategy. The four sources used were selected because PubMed and Europe PMC together cover MEDLINE and PubMed Central with full-text indexing of open-access material, while OpenAlex and Semantic Scholar index records beyond MEDLINE and provide the citation graphs used for forward searching; empirical comparisons of database combinations indicate that a combination comprising MEDLINE, a full-text source, and a broad multidisciplinary index retrieves the large majority of includable records for clinical questions, although no combination is complete and Embase in particular adds materially in some fields [34]. Because a substantial proportion of positive MTHFR findings has been reported from East Asian cohorts, the absence of Chinese-language databases is a specific risk: reports published only in Chinese-language journals that are not indexed in the four sources searched could not be retrieved, which may bias the synthesis towards internationally indexed, English-language reports and towards the studies that such reports cite. Nine of the thirteen included studies were nevertheless conducted in East Asia, and forward citation searching of every included report together with reference checking of three related meta-analyses that did search Chinese-language sources [17,25,26] yielded no additional eligible reports; this limits, but does not exclude, the possibility of unretrieved eligible studies. No language restriction was applied to the records that were retrieved.

### 2.4. Selection Process

Records were de-duplicated by PubMed identifier, digital object identifier, and normalised title. A deterministic, rule-based triage was then applied to titles, abstracts, and indexing terms to remove records that were clearly ineligible: reviews, meta-analyses, editorials, comments, case reports, guidelines, retractions, and errata; animal-only studies; records lacking any imaging concept; records lacking any cognitive or cerebrovascular concept; and, for Europe PMC full-text matches, records in which no MTHFR or homocysteine pathway term appeared in the title, abstract, or indexing terms. The rules and their outputs were archived so that every automated exclusion could be traced. To guard against over-exclusion, the reviewer also inspected the titles of all automation-excluded records and re-instated two for full-text assessment. All remaining records were screened by the reviewer on title and abstract, and potentially eligible reports were retrieved and assessed in full text against the eligibility criteria. The triage was deliberately deterministic and rule-based: no machine learning classifier was used to rank or to exclude records because the performance of text mining approaches to study identification varies substantially between reviews and such tools are generally recommended as an adjunct to human screening, with explicit safeguards against over-exclusion, rather than as a replacement for it [35]. The safeguard adopted here was manual inspection of the title of every automation-excluded record. Screening was then performed by a single reviewer. Independent duplicate screening is the methodological reference standard, and comparative studies show that single screening misses a small but non-negligible proportion of includable records [36]; the single-reviewer design reflects the resources available for this review and is acknowledged as a limitation. Its likely impact was reduced by the deliberately sensitive PubMed strategy, the manual title check of all automation-excluded records, forward citation searching of every included report, and reference list checking of included reports and related meta-analyses.

### 2.5. Data Collection and Data Items

Data were extracted from the full text, tables, figures, and Supplementary Files of each included report into a structured form. Data items comprised country and setting, recruitment, design, diagnostic criteria, sample size with genotype and MRI, genotype counts, age, sex, genotyping method, genetic model, MRI field strength and sequences, image analysis method, structural outcomes, covariates, homocysteine and folate measurements, cognitive correlates, and follow-up. Potential confounders of the genotype–imaging association were recorded explicitly as reported by each study: age, sex, hypertension and other vascular risk factors, diabetes, education, APOE genotype, renal function, homocysteine, folate and vitamin B12 status, and the ancestry or country of the cohort. The covariates that entered the genotype contrast in each study are tabulated in the Results (Section 3.3). No included study adjusted the genotype–imaging contrast for renal function, and none adjusted for the full set; because the reports provide aggregate data only, these variables could not be controlled in the present synthesis, a constraint that governs the interpretation of the pooled estimates (Section 2.7) and is revisited in the Discussion. Numerical contrasts were checked against the original tables and supplements; where a report gave only percentages, counts were reconstructed from the stated denominators. Unavailable genotype-specific statistics are marked as not reported (NR); no values were imputed from related studies or estimated from plots. Companion reports were linked at cohort level: the two Alzheimer’s Disease Neuroimaging Initiative (ADNI) analyses were not treated as independent replication, and possible overlap between reports from the same Korean and Anhui clinics could not be resolved, so sample sizes are presented by report, and no exact total of unique participants is asserted.

### 2.6. Quality Appraisal

Study quality was appraised with an adapted Newcastle–Ottawa Scale (NOS) [37]. Adaptation was necessary because the original scale was developed for case–control and cohort studies of a dichotomous exposure and outcome and contains items—ascertainment of exposure, non-response rate, adequacy of follow-up—that do not map onto a cross-sectional genotype–imaging contrast while omitting the features that most threaten validity in imaging genetics: the size of the genotype subgroups, genotyping quality control, and completeness of genotype-specific reporting. Items were therefore rewritten in line with the STREGA reporting recommendations for genetic association studies [32] while retaining the NOS selection–comparability–outcome architecture, and the adapted scale was scored on nine equally weighted items: four selection items (clearly defined or consecutive sampling frame; at least 100 genotyped and scanned participants with at least 20 TT homozygotes; validated genotyping assay with quality control or Hardy–Weinberg testing; established diagnostic criteria for the cognitive disorder), two comparability items (age and sex accounted for in the genotype–imaging analysis; additional confounders such as education, APOE, vascular risk factors, or homocysteine accounted for), and three outcome items (blinded rating or automated image analysis; multiple comparison control or a prespecified primary contrast with a stated genetic model; complete genotype-specific reporting with effect sizes and uncertainty). Scores of 7–9, 4–6, and 0–3 were interpreted as low, moderate, and high concern, respectively, bearing in mind the recognised limitations of summary scores [38]. The trial-embedded genotype analysis in VITACOG was scored on the observational genotype contrast rather than on the trial’s randomised comparison.

### 2.7. Synthesis Methods

Findings were grouped by clinical population (AD, MCI, symptomatic CSVD, mixed cognitively impaired cohorts) and by structural outcome domain (regional volume or cortical atrophy, hippocampal or medial temporal measures, global atrophy rate, white matter lesion burden, lacunes, microbleeds, and diffusion measures), and genotype main effects were distinguished from interactions, mediation analyses, and longitudinal findings. An evidence map summarises the direction of every genotype–outcome comparison. Exploratory random-effects meta-analyses were performed only where at least three studies reported comparable, unadjusted, genotype-stratified data on the same outcome. Two such data sets existed: moderate-to-severe white matter lesion burden (Fazekas or Age-Related White Matter Changes grade 2–3, or confluent white matter changes) tabulated by genotype, and plasma total homocysteine by genotype. Odds ratios were pooled under dominant (CT + TT vs. CC), recessive (TT vs. CT + CC), and homozygote (TT vs. CC) models with the DerSimonian–Laird random-effects estimator [39], and heterogeneity was quantified with Cochran’s Q and I^2^ [40]. That estimator was chosen for transparency and for comparability with the earlier MTHFR meta-analyses against which these results will be read [16,25,26], while recognising that it underestimates the between-study variance and yields confidence intervals that are too narrow when few studies are pooled, and that likelihood-based estimators or the Hartung–Knapp–Sidik–Jonkman adjustment are preferable in that situation [41,42]. Because only three to six studies contributed to any single analysis, the intervals reported here should be regarded as optimistically narrow. Mean differences in homocysteine were pooled for T-allele carriers versus CC and for TT versus CC; medians with interquartile ranges were converted to means and standard deviations following the method of Wan et al. [43], and a report giving only standard errors that were internally inconsistent was excluded from this analysis. Voxel-wise, whole-brain atrophy rate, hippocampal subfield, and adjusted regression results were not pooled because they did not share a common effect measure. Analyses were implemented in Python 3.11 (Python Software Foundation, Wilmington, DE, USA) with NumPy 2.4 and SciPy 1.17 (open-source libraries, https://numpy.org and https://scipy.org), and the input data and code are available from the corresponding author upon reasonable request. Because the pooled estimates are unadjusted and rest on few studies, they are presented as descriptive summaries rather than as confirmatory tests. In particular, the pooled lesion estimates combine unadjusted counts and are therefore uncontrolled for age, hypertension and other vascular risk factors, renal function, homocysteine, folate status, and ancestry, each of which is associated with white matter lesion burden and, in the case of ancestry, with genotype frequency; they cannot distinguish an independent effect of C677T from confounding by these variables. Confounder-adjusted pooling was not possible because the included reports did not provide a common adjusted effect measure, and the adjusted estimates that individual studies did report are therefore tabulated separately from the crude estimates in the Results (Section 3.4) so that the two are not conflated. Whether each study applied a correction for multiple comparisons, and whether a positive result arose from a prespecified primary contrast or from a subgroup or interaction analysis, was recorded as an explicit data item; this information is reflected in the outcome domain of the quality appraisal and in the evidence map, in which findings limited to an interaction or a subgroup are distinguished from genotype main effects.

## 3. Results

### 3.1. Study Selection

The searches identified 3138 records (PubMed 722, Europe PMC 2203, OpenAlex 112, Semantic Scholar 101). After removal of 387 duplicates, 2594 records marked ineligible by the rule-based triage, and one non-article record, 156 records were screened by title and abstract by the reviewer, and 137 were excluded. Nineteen reports were sought and retrieved in full and assessed for eligibility; six were excluded because the population was not an eligible cognitive cohort (an imaging-defined white matter lesion series that excluded cognitive disorders [44] and a lacunar stroke cohort without cognitive assessment [45]), because the exposure was not C677T-specific (a polygenic homocysteine score without a single-variant imaging contrast [46] and a diffusion study that did not genotype MTHFR [47]), because structural MRI was not reported by genotype [48], or because the report was a narrative perspective without a comparative genotype–imaging analysis [49]. Thirteen reports of thirteen studies were included [20,50,51,52,53,54,55,56,57,58,59,60,61]; forward citation searching and reference checking identified no additional eligible reports, and the only study not indexed in PubMed (a Korean AD cohort in Neurology Asia [52]) was identified through OpenAlex and Semantic Scholar. Figure 1 shows the PRISMA 2020 flow diagram.

### 3.2. Study Characteristics

Table 1 summarises the 13 included studies, which enrolled 3880 participants with genotype and MRI data across reports; because the two ADNI analyses share participants [51,54] and two pairs of reports originate from the same Korean [55,57] and Anhui [58,60] clinics, the number of unique participants is smaller than this total and cannot be determined from the published reports. The figure of 3880 is a sum of report-level sample sizes and must not be read as an independent sample size; participant-level de-duplication would require individual participant data or cohort identifiers that the reports do not provide. Retaining only the larger report of each overlapping pair and discarding the smaller gives a conservative lower bound of approximately 2900 participants, but even this figure assumes complete overlap within each pair and complete independence between centres, neither of which can be verified. No pooled analysis in this review includes more than one report from any overlapping pair, so the meta-analytic estimates are not inflated by double counting. Five studies examined AD [52,53,56,57,60], three MCI [20,51,58], three symptomatic CSVD [50,59,61], and two mixed cognitively impaired cohorts [54,55]. Nine studies were conducted in East Asia (five in mainland China, one in Taiwan, three in South Korea), two in Europe (Serbia and the United Kingdom), and two in the United States. Sample sizes with genotype and MRI ranged from 60 to 819, and the number of TT homozygotes ranged from six to 141. The T-allele frequency ranged from 25% in the Taiwanese AD cohort to 43% in the Anhui MCI cohort, and mean age from 60 years in the Serbian CSVD series to 79 years in the Guangzhou CSVD series. Imaging was cross-sectional in eleven studies; Rajagopalan et al. reported 12-month follow-up in ADNI [51] and the VITACOG genotype analysis used serial MRI over 24 months [20]. Field strength was 1.0 T in one study, 1.5 T in five, and 3.0 T in seven.

### 3.3. Genotyping, Imaging Methods, and Homocysteine by Genotype

Table 2 lists the genetic models, imaging methods, covariate adjustment, and genotype-specific homocysteine concentrations. Four studies used a dominant contrast (T carriers versus CC), two a recessive contrast (TT versus C-allele carriers), two an additive allele dose model, and five a three-genotype comparison, in one case with an additional allelic comparison [56] and in another with a regression coefficient labelled as a genotype term without a fully specified coding [59]. Structural outcomes were visual white matter scales in seven studies, voxel-wise or region-of-interest volumetry in four, whole-brain atrophy rate in one, hippocampal subfield segmentation in one, diffusion tensor imaging in one, and microbleed or lacune assessments in five. Covariate adjustment of the genotype contrast ranged from none (three studies) to multivariable models including vascular risk factors and homocysteine. Where genotype-specific homocysteine values were reported, T-allele carriers and particularly TT homozygotes had higher plasma concentrations in every study, with mean or median values in TT homozygotes between 11.7 and 24.1 µmol/L.

### 3.4. Structural MRI Findings by Population and Outcome

Table 3 presents the principal genotype contrasts with effect estimates. In MCI, the ADNI analysis reported regional white matter volume deficits per T allele in bilateral periventricular fronto-parietal white matter at baseline and accelerated regional white matter atrophy over 12 months, with replication of a posterior white matter deficit in the small Cardiovascular Health Study sample but no association with WMH volume [51]. The VITACOG trial found no genotype effect on the whole-brain atrophy rate and no genotype-by-treatment interaction [20]. In the Anhui amnestic MCI cohort, genotype had no main effect on grey matter volume; a diagnosis-by-genotype interaction in the right precuneus was confined to APOE ε4 non-carriers and did not survive correction [58].

In AD, the Taiwanese cohort reported a smaller posterior cingulate seed volume in T carriers but no difference in three other seed regions or in any of 11 white matter tracts on diffusion imaging [53]. The Changsha sub-cohort reported a shift towards higher Fazekas grades with the T allele [56], whereas the Korean cohorts found no association of genotype with WMH severity [52] or with medial temporal atrophy, cortical atrophy, white matter lesions, or microbleeds [57]. Hippocampal subfield volumes did not differ between AD T carriers and CC homozygotes after correction; lower volumes of the right dentate gyrus granule cell layer, CA3, and CA4 heads were confined to the combined low-folate/T-carrier stratum [60]. In the diagnosis-adjusted ADNI cohort, each T allele was associated with lower volume in cingulate, frontal, orbitofrontal, parietal, temporal, thalamic, and periventricular regions, and the medial orbitofrontal association was partially mediated by homocysteine [54].

In symptomatic CSVD, the Fuan cohort reported higher adjusted odds of any WMH for TT than for CC homozygotes, with a significant allele dose trend, and no genotype difference in microbleeds or lacunes [61]. The Guangzhou cohort reported a genotype-dependent distribution of ARWMC grades that persisted after adjustment, and no association with lacunar infarction [59]. The Serbian series found no significant association between TT homozygosity and confluent white matter changes, while homocysteine itself was an independent predictor [50]. In the Korean memory clinic registry, TT homozygosity was somewhat more frequent in patients with microbleeds and, among those with microbleeds, in patients with lobar rather than exclusively deep lesions, but genotype was not entered in the multivariable model [55]. Effect estimates, confidence intervals, and *p* values for all of these contrasts are given in Table 3.

### 3.5. Pooled Analyses

Five studies tabulated a comparable white matter lesion outcome by genotype (Figure 2). Under the recessive model, TT homozygotes had higher unadjusted odds of moderate-to-severe white matter lesions than C-allele carriers in all four contributing studies, with no detectable heterogeneity, and the three CSVD studies alone gave a closely similar estimate. The homozygote contrast pointed in the same direction with a larger magnitude. The dominant model, by contrast, was heterogeneous and non-significant because the Korean and Fuan cohorts showed no excess lesion burden in heterozygotes, whereas the Changsha and Guangzhou cohorts did, and the two AD studies contributing to this model pointed in opposite directions. Pooled values, confidence intervals, and heterogeneity statistics are given in Table 3 and Figure 2. All of these estimates are unadjusted and rest on three to four studies.

Six studies reported homocysteine by genotype in a form that could be pooled (Figure 3). Plasma total homocysteine was higher in T-allele carriers than in CC homozygotes, and higher still in TT homozygotes, with the largest differences in the Chinese cohorts [56,61]; pooled mean differences and heterogeneity statistics are given in Figure 3 and Table 3. The genotype–homocysteine gradient was present in every study in which homocysteine was measured, including those whose imaging findings were null.

**Table 1:** Characteristics of the included studies and of the samples with genotype and structural MRI data.

Study [Ref]	Country	Population	Design	n with Genotype + MRI	Genotype CC/CT/TT (n)	T-allele Frequency (%)	Age, Years (Mean ± SD)	Female (%)	MRI Field (T)
Smith 2010 (VITACOG) [20]	United Kingdom	MCI	RCT genotype analysis; L (24 mo)	168	68/83/17	34.8	76.6 ± 4.9	60.7	1.5
Pavlovic 2011 [50]	Serbia	Symptomatic CSVD (VCI 35%, VaD 11%)	CS, consecutive	95	36/42/17	40.0	59.8 ± 10.9	42.1	1.0
Rajagopalan 2012 [51]	United States	Amnestic MCI	CS + L (12 mo); replication cohort	359	149/157/53	36.6	75.0 ± 7.1	35.7	1.5
Jeong 2015 [52]	South Korea	Probable AD	CS, retrospective	75	28/47 (T carriers)	NR (carriers 62.7)	71.9 ± 6.5	73.3	1.5
Chang 2017 [53]	Taiwan	AD (CDR 0.5–1)	CS	135	73/56/6	25.2	73.4 ± 7.6	51.9	3.0
Roussotte 2017 [54]	United States	AD 173/MCI 359/CN 206	CS, diagnosis-adjusted	738	314/320/104	35.8	75.5 ± 6.8	40.7	1.5
Yoo 2020 [55]	South Korea	Memory clinic patients (subtype NR)	CS, retrospective registry	819	678 (CC + CT)/141	NR (TT 17.2)	74.1 ± 9.7	69.2	3.0
Jiang 2021 [56]	China	Probable/definite AD	CS imaging sub-cohort of case–control study	121	57/54/10	30.6	65.8 ± 10.9	59.1	3.0
Kim 2021 [57]	South Korea	Probable AD	Retrospective cohort; CS imaging	574	NR	NR	73.2	72.0	3.0
You 2021 [58]	China	Amnestic MCI	CS case–control	60	20/28/12	43.3	67.3 ± 9.4	55.0	3.0
Li 2022 [59]	China	Symptomatic CSVD (>65 y)	CS, admitted patients	304	127/118/59	38.8	79.3 ± 8.8	42.4	1.5
Tang 2024 [60]	China	MCI due to AD + AD dementia	CS case–control	102	34/68 (T carriers)	NR (carriers 66.7)	63.5 (median)	52.0	3.0
Wang 2024 [61]	China	CSVD (STRIVE); CI in 56%	CS, retrospective	330	149/112/69	37.9	64.0 (median)	45.8	3.0

AD, Alzheimer’s disease; CDR, Clinical Dementia Rating; CI, cognitive impairment; CN, cognitively normal; CS, cross-sectional; CSVD, cerebral small vessel disease; L, longitudinal; MCI, mild cognitive impairment; mo, months; NR, not reported; RCT, randomised controlled trial; STRIVE, Standards for Reporting Vascular Changes on Neuroimaging; VaD, vascular dementia; VCI, vascular cognitive impairment. Genotype counts and T-allele frequency refer to the sample with genotype and MRI data. For Rajagopalan et al., the ADNI MCI sample is shown; the Cardiovascular Health Study replication sample comprised 51 participants (CC 16, CT 29, TT 6). For Jiang et al., age and sex refer to the whole AD cohort (n = 721) because the imaging sub-cohort was not described separately. For Smith et al., age and sex were pooled from the two trial arms. For Jeong et al., the genotype table in the source is internally inconsistent (n = 61 versus 75); the carrier split from the outcome table (n = 75) is used. Age for Wang et al. and Tang et al. is a median. The two ADNI reports overlap and samples must not be summed.

**Table 2:** Genotyping, genetic model, structural MRI methods, covariate adjustment, and plasma total homocysteine by genotype.

Study [Ref]	Genotyping	Genetic Model	Structural MRI Outcome and Analysis	Adjustment in Genotype Analysis	tHcy CC, µmol/L (n)	tHcy CT or T Carriers (n)	tHcy TT (n)	tHcy Comparison
Smith 2010 (VITACOG) [20]	Amplifluor SNP assay	Three-genotype (ANOVA)	SIENA whole-brain atrophy rate, 2 scans 24 mo apart	Age; treatment arm (ANOVA)	—	—	—	NR by genotype
Pavlovic 2011 [50]	PCR-RFLP (HinfI)	Recessive (TT vs. CT + CC)	ARWMC visual scale; confluent WMCs; lacunes	Age, sex, disease duration	12.8 ± 4.9 (36)	15.4 ± 4.2 (42)	14.9 ± 6.5 (17)	ANOVA *p* = 0.066
Rajagopalan 2012 [51]	Illumina 610-Quad; PCR (CHS)	Additive (T-allele dose)	Tensor-based morphometry (FDR q 0.05); FreeSurfer WM volume; WMH	Age, sex (APOE, homocysteine, folate in sensitivity models)	10.2 (149)	10.4 (157)	11.7 (53)	means (SE) only; *p* < 0.0001
Jeong 2015 [52]	PCR (reference laboratory); HWE *p* > 0.05	Dominant (T carriers vs. CC)	CREDOS WMH scale (mild/moderate/severe), two blinded raters	None (chi-square)	—	—	—	not measured
Chang 2017 [53]	Sequenom MALDI-TOF	Dominant (T carriers vs. CC)	VBM seed volumes + structural covariance; DTI 11 tracts	Groups matched on age, sex, education, APOE	11.4 ± 3.6 (73)	14.0 ± 4.8 (62) [T carriers]	—	*p* = 0.001
Roussotte 2017 [54]	Illumina 610-Quad	Additive (T-allele dose); recessive in supplement	Tensor-based morphometry (searchlight FDR); FreeSurfer medial OFC volume	Age, sex, diagnosis	—	—	—	values NR; F = 10.4, *p* < 0.001
Yoo 2020 [55]	Real-time PCR kit	Recessive (TT vs. non-TT)	Microbleeds on T2*-GRE (presence, count, location); Fazekas WMH	None for genotype (multivariable model excluded genotype)	—	—	—	values NR; TT > CC/CT (figure)
Jiang 2021 [56]	Sanger sequencing	Allelic and three-genotype (unadjusted)	Fazekas scale (0–3) on 3D FLAIR	None	14.0 ± 3.9 (57)	14.4 ± 5.0 (54)	24.1 ± 15.6 (10)	TT vs. CC *p* = 0.036
Kim 2021 [57]	PCR-RFLP (HinfI); HWE confirmed	Three-genotype and T-allele dose (ANCOVA); sex-stratified	Fazekas (deep, periventricular), Scheltens MTA, Pasquier cortical atrophy, microbleeds (SWI)	Age, sex (folate for homocysteine)	—	—	—	values NR; TT > CC, *p* = 0.005
You 2021 [58]	Penta-primer ARMS-PCR; HWE confirmed	Dominant (T carriers vs. CC)	VBM8/DARTEL GM volume, full-factorial ANCOVA (cluster FWE *p* < 0.05)	Age, sex, education, TIV	—	—	—	NR by genotype
Li 2022 [59]	Microarray hybridisation PCR kit	Three-genotype; regression term for TT genotype	ARWMC scale (0–3), two raters; lacunar infarction	Sex, age, CHD, lacunar infarction, homocysteine	16.3 ± 5.1 (127)	17.5 ± 6.3 (118)	20.3 ± 8.2 (59)	*p* < 0.001
Tang 2024 [60]	PCR and sequencing	Dominant (T carriers vs. CC); folate × genotype risk strata	FreeSurfer 7.2 hippocampal subfields (FDR); ASL perfusion	Sex, age, education, APOE	16.2 (34)	17.6 (68) [T carriers]	—	medians; *p* = 0.029
Wang 2024 [61]	PCR with published primers	Three-genotype (CC reference) with trend test	Fazekas scale (0–3), two neurologists; microbleeds (SWI); lacunes	Age category, hypertension, hyperhomocysteinaemia	10.1 (149)	13.2 (112)	18.1 (69)	medians; *p* < 0.001

Values are mean ± SD unless stated; Rajagopalan et al. reported means with standard errors (CC 10.2, CT 10.4, TT 11.7); Tang et al. and Wang et al. reported medians. ANCOVA, analysis of covariance; ARMS, amplification refractory mutation system; ARWMCs, Age-Related White Matter Changes; ASL, arterial spin labelling; CHD, coronary heart disease; CREDOS, Clinical Research for Dementia of South Korea; DARTEL, Diffeomorphic Anatomical Registration Through Exponentiated Lie Algebra; DTI, diffusion tensor imaging; FDR, false discovery rate; FLAIR, fluid-attenuated inversion recovery; FWE, family-wise error; GM, grey matter; GRE, gradient-recalled echo; HWE, Hardy–Weinberg equilibrium; MALDI-TOF, matrix-assisted laser desorption/ionisation time-of-flight; MTA, medial temporal atrophy; NR, not reported; OFC, orbitofrontal cortex; PCR, polymerase chain reaction; RFLP, restriction fragment length polymorphism; SIENA, Structural Image Evaluation using Normalisation of Atrophy; SWI, susceptibility-weighted imaging; tHcy, plasma total homocysteine; TIV, total intracranial volume; VBM, voxel-based morphometry; WM, white matter; WMCs, white matter changes; WMHs, white matter hyperintensities.

**Table 3:** Principal genotype contrasts for structural MRI outcomes, with effect estimates, and pooled estimates from the exploratory meta-analyses.

Study [Ref]	Population	Outcome	Contrast	Effect Estimate (95% CI) or Genotype Values	Statistical Result	Direction
Smith 2010 [20]	MCI	Whole-brain atrophy rate (%/y), placebo arm	CC/CT/TT	1.03/1.10/1.23	ANOVA *p* = 0.947; genotype × treatment *p* = 0.577	Null
Pavlovic 2011 [50]	CSVD	Confluent WMCs present	TT vs. CC + CT	Crude OR 1.67 (0.54–5.20); adjusted OR 0.6 (0.2–1.8) *	*p* = 0.340	Null
Rajagopalan 2012 [51]	MCI	Regional WM volume (TBM), per T allele	Additive	Up to −6% (ADNI); up to −12% (CHS)	FDR q = 0.05	T allele: lower volume
		Annual regional atrophy (12 mo), per T allele	Additive	Up to −1.5%/y	FDR q = 0.05	T allele: faster atrophy
		Cerebral WM volume (FreeSurfer)	Additive	Left *p* = 0.02, right *p* = 0.046 (baseline)	—	T allele: lower volume
		WMH volume	Additive	—	*p* = 0.54	Null
Jeong 2015 [52]	AD	Moderate-to-severe WMHs (CREDOS)	CT + TT vs. CC	16/47 vs. 13/28; crude OR 0.60 (0.23–1.55)	χ^2^ *p* = 0.50	Null
Chang 2017 [53]	AD	Posterior cingulate seed GM volume	CT + TT vs. CC	Smaller in T carriers	*p* < 0.05	T allele: lower volume
		Entorhinal, frontoinsular, DLPFC seed volumes	CT + TT vs. CC	No difference	*p* > 0.05	Null
		DTI in 11 tracts (FA, MD, AxD, RD)	CT + TT vs. CC	No difference (e.g., forceps major FA 0.48 ± 0.04 vs. 0.47 ± 0.04)	*p* > 0.05	Null
Roussotte 2017 [54]	Mixed	Regional volume (TBM), per T allele	Additive	−2 to −4%	Searchlight FDR q = 0.05	T allele: lower volume
		Medial orbitofrontal volume (ROI, n = 640)	Additive	F = 3.428	*p* = 0.033	T allele: lower volume
		Mediation by homocysteine (indirect effect)	Additive	−0.015 (−0.039 to −0.003); 25% of total effect	Bootstrap CI excludes 0	Partial mediation
Yoo 2020 [55]	Mixed	Cerebral microbleeds present	TT vs. CC + CT	36/161 vs. 105/658; crude OR 1.52 (0.99–2.32)	*p* = 0.055	Null
		Lobar vs. deep-only microbleeds (TT frequency)	TT vs. CC + CT	25/88 (28.4%) vs. 11/73 (15.1%)	*p* = 0.043	TT: more lobar (unadjusted)
Jiang 2021 [56]	AD	Fazekas 2–3	TT vs. CC	7/10 vs. 16/57; crude OR 5.98 (1.37–26.02)	Genotype *p* = 0.024; allele *p* = 0.006	T allele: more WML
		Fazekas 2–3	CT + TT vs. CC	37/64 vs. 16/57; crude OR 3.51 (1.64–7.52)	—	T allele: more WML
Kim 2021 [57]	AD	MTA, cortical atrophy, deep/periventricular WMLs, microbleeds	Three-genotype; allele dose	NR (not significant)	*p* > 0.05	Null
You 2021 [58]	MCI	GM volume (VBM), main effect	CT + TT vs. CC	No cluster	Cluster FWE *p* < 0.05	Null
		Diagnosis × genotype, right precuneus (APOE ε4 non-carriers)	CT + TT vs. CC	81 voxels; F = 15.55	*p* < 0.001 uncorrected	Interaction only
Li 2022 [59]	CSVD	ARWMCs 2–3	TT vs. CC	36/59 vs. 52/127; crude OR 2.26 (1.20–4.25)	Distribution Z = 16.05, *p* = 0.001	T allele: more WML
		ARWMCs 2–3, adjusted	Genotype term (TT) †	OR 1.54 (1.05–2.28)	*p* = 0.029	T allele: more WML
		Lacunar infarction	CC/CT/TT	35.4%/35.6%/39.0%	*p* = 0.883	Null
Tang 2024 [60]	AD	Hippocampal subfield volumes (19 per hemisphere)	CT + TT vs. CC	No difference after FDR	FDR q = 0.05	Null
		Right DG-GCL, CA3, CA4 head volumes	Low folate + T allele vs. lower-risk strata	Lower in high-risk stratum	*p* < 0.05 (uncorrected across strata)	Subgroup only
Wang 2024 [61]	CSVD	Any WMHs (Fazekas ≥ 1), adjusted	TT vs. CC; CT vs. CC	OR 2.71 (1.10–6.69); OR 1.72 (0.97–3.06)	*p* = 0.031; *p* = 0.064; trend *p* = 0.041	T allele: more WMH
		Fazekas 2–3	TT vs. CC	34/69 vs 58/149; crude OR 1.52 (0.86–2.71)	—	Null (crude)
		Microbleeds; lacunes	CC/CT/TT	30.9/22.3/26.1%; 26.2/24.1/30.4%	*p* = 0.300; *p* = 0.642	Null
Pooled (four studies) [50,56,59,61]	AD + CSVD	Moderate-to-severe WMLs, recessive model	TT vs. CC + CT	1.65 (1.15–2.36)	*p* = 0.006; I^2^ = 0%	TT: more WML
Pooled (three studies) [56,59,61]	AD + CSVD	Moderate-to-severe WMLs, homozygote contrast	TT vs. CC	2.12 (1.22–3.69)	*p* = 0.008; I^2^ = 36%	TT: more WML
Pooled (four studies) [52,56,59,61]	AD + CSVD	Moderate-to-severe WMLs, dominant model	CT + TT vs. CC	1.57 (0.83–2.96)	*p* = 0.161; I^2^ = 77%	Inconclusive
Pooled (six studies) [50,53,56,59,60,61]	AD + CSVD	Plasma tHcy, mean difference (µmol/L)	CT + TT vs. CC	2.80 (2.09–3.52)	*p* < 0.001; I^2^ = 22%	T allele: higher tHcy
Pooled (four studies) [50,56,59,61]	AD + CSVD	Plasma tHcy, mean difference (µmol/L)	TT vs. CC	5.12 (2.29–7.96)	*p* < 0.001; I^2^ = 75%	TT: higher tHcy

Crude odds ratios were calculated from the reported counts (events/n in the T-allele group versus the reference group) and are unadjusted; adjusted estimates are as reported by the authors. * The direction and coding of the adjusted odds ratio reported by Pavlovic et al. could not be reconciled with the tabulated counts (TT in 12/58 with and 5/37 without confluent changes). † The regression term in Li et al. is labelled ‘MTHFR genotype’ in the model table and ‘TT genotype’ in the text; the reference category is not fully specified. ARWMCs, Age-Related White Matter Changes; AxD, axial diffusivity; CA, cornu ammonis; CHS, Cardiovascular Health Study; CI, confidence interval; CREDOS, Clinical Research for Dementia of South Korea; DG-GCL, dentate gyrus granule cell layer; DLPFC, dorsolateral prefrontal cortex; DTI, diffusion tensor imaging; FA, fractional anisotropy; FDR, false discovery rate; FWE, family-wise error; GM, grey matter; MD, mean diffusivity; MTA, medial temporal atrophy; NR, not reported; OR, odds ratio; RD, radial diffusivity; ROI, region of interest; TBM, tensor-based morphometry; tHcy, plasma total homocysteine; WM, white matter; WMCs, white matter changes; WMHs, white matter hyperintensities; WMLs, white matter lesions. Pooled estimates are DerSimonian–Laird random-effects estimates from unadjusted data.

**Figure 2:**
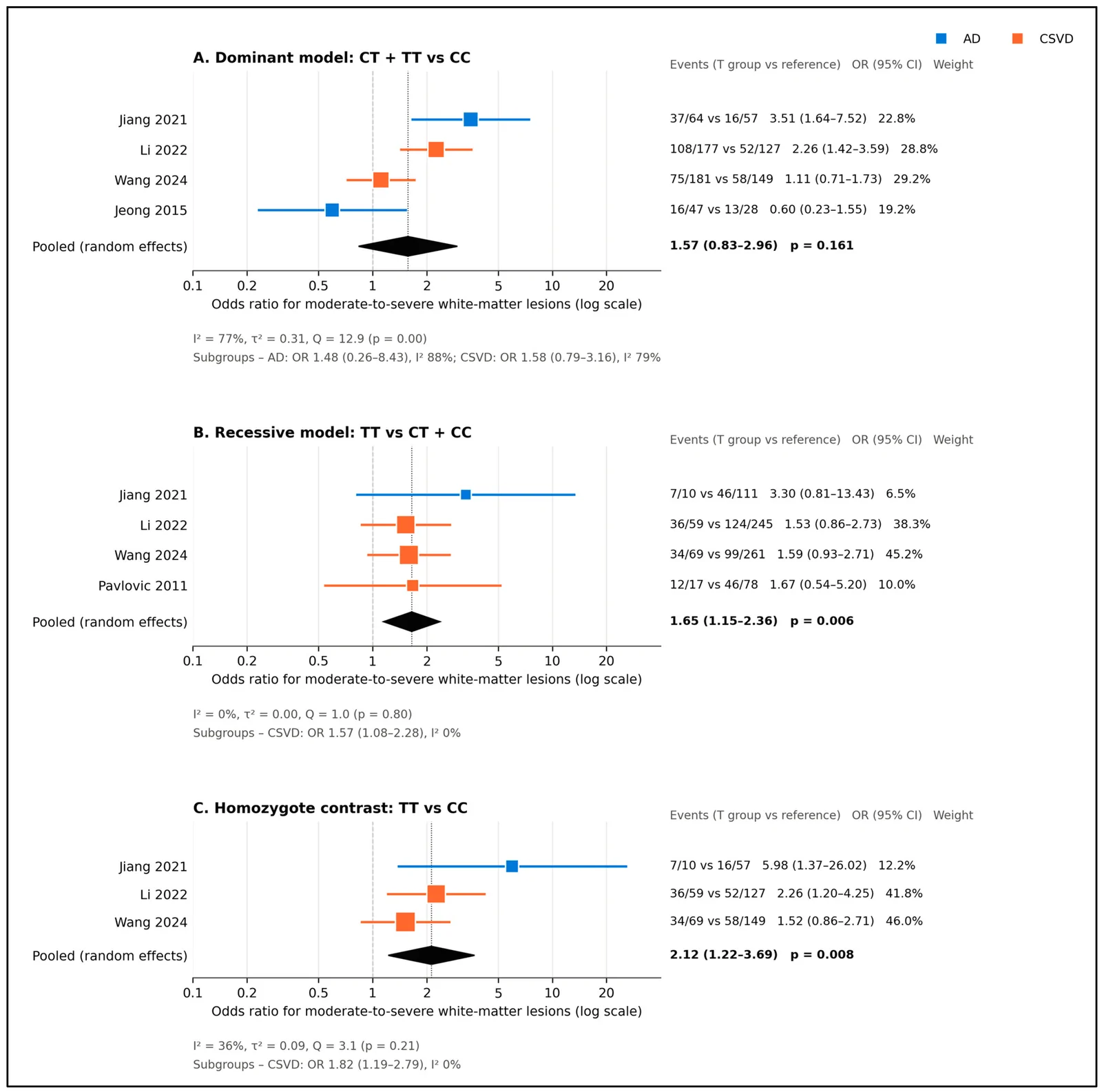
Random-effects meta-analysis of moderate-to-severe white matter lesion burden (Fazekas or Age-Related White Matter Changes grade 2–3, or confluent white matter changes) by MTHFR C677T genotype under (**A**) dominant, (**B**) recessive, and (**C**) homozygote contrasts. Squares are study odds ratios (area proportional to random-effects weight), horizontal lines are 95% confidence intervals, and diamonds are pooled DerSimonian–Laird estimates. Counts are events/n in the T-allele group versus the reference group and are unadjusted. AD, Alzheimer’s disease; CSVD, cerebral small vessel disease.

### 3.6. Evidence Map and Methodological Quality

Figure 4 shows that positive genotype associations cluster in two domains: voxel-wise regional volume in the ADNI-derived cohorts and visual white matter lesion grades in Chinese AD and CSVD cohorts. Every comparison involving hippocampal or medial temporal measures, global atrophy rate, lacunes, microbleeds, or diffusion metrics was null or confined to an uncorrected subgroup or interaction. In contrast, the homocysteine column is uniformly positive wherever it was measured.

**Figure 3:**
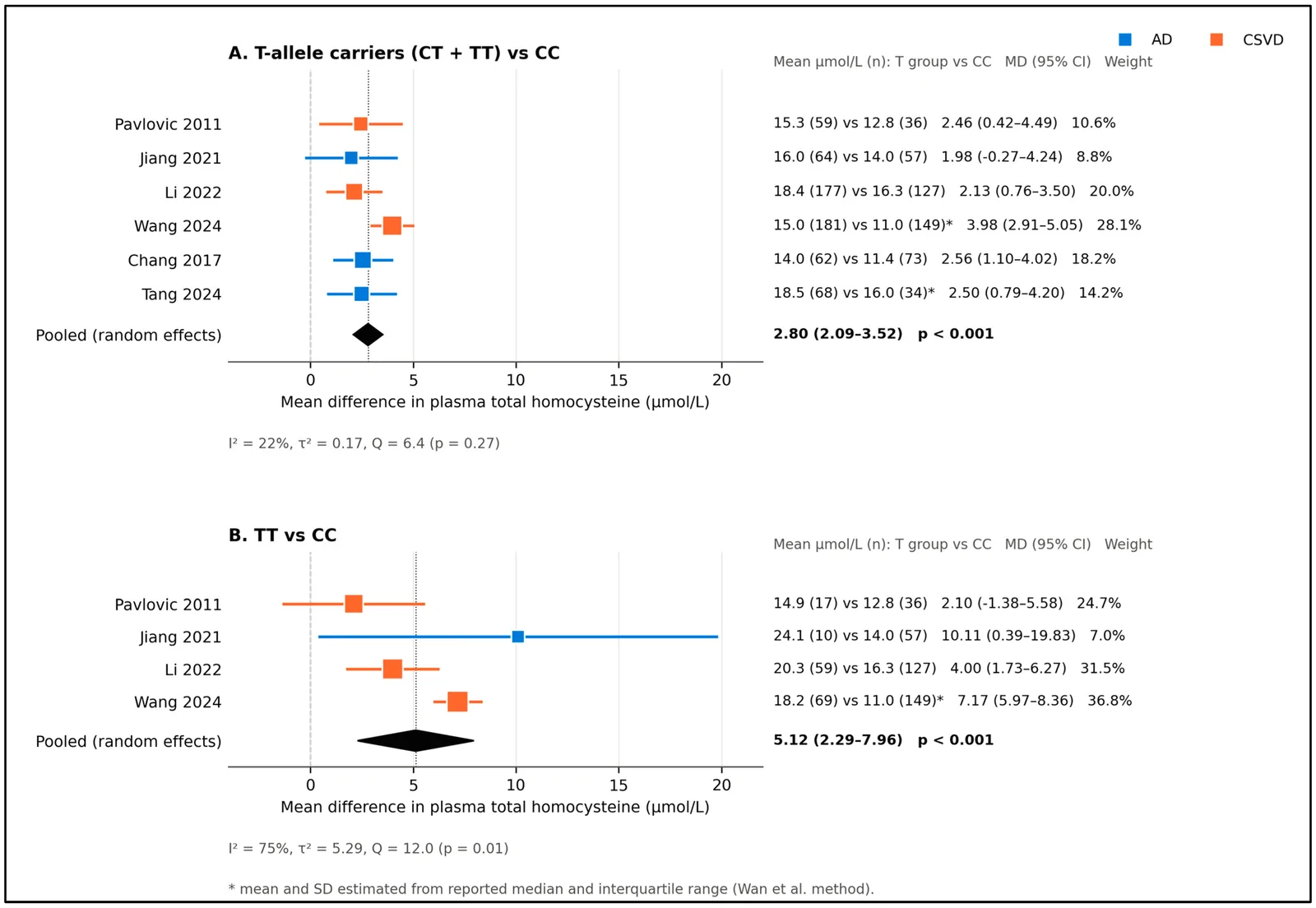
Random-effects meta-analysis of plasma total homocysteine (µmol/L) by MTHFR C677T genotype: (**A**) T-allele carriers versus CC; (**B**) TT versus CC. Means and standard deviations for Wang et al. [61] and Tang et al. [60] were estimated from medians and interquartile ranges. Rajagopalan et al. [51] were excluded because only standard errors with an internally inconsistent TT value were reported. AD, Alzheimer’s disease; CSVD, cerebral small vessel disease; MD, mean difference.

Adapted NOS totals ranged from 3 to 9 (median 6; Table 4). Four studies were rated as low concern (scores 7–9), eight as moderate concern (4–6), and one as high concern (3). The most frequent deficits were small TT subgroups (fewer than 20 TT homozygotes in five studies and an unreported TT count in three), absence of adjustment for age and sex in the genotype–imaging analysis (three studies), incomplete genotype-specific reporting with effect sizes and uncertainty (seven studies), and analyses without multiple comparison control or a prespecified genetic model (seven studies). Only the diagnosis-adjusted ADNI analysis and the Fuan CSVD study satisfied all three outcome items. Correction for multiple comparisons was applied inconsistently across the evidence base. Four studies controlled the false discovery rate or the family-wise error rate across their primary imaging analyses [51,54,58,60], whereas the remaining nine reported multiple regions, lesion subtypes, or genetic models without any stated correction. Several of the positive findings were observed only within a subgroup or an interaction analysis, or were obtained without correction, and are identified as such in Table 3 and Figure 4: these include the precuneus interaction restricted to APOE ε4 non-carriers [58], the folate-stratified hippocampal subfield findings [60], the lobar-versus-deep microbleed comparison [55], and the single-seed posterior cingulate difference [53]. They are treated throughout this review as preliminary and are not counted as independent confirmations of a genotype effect.

**Figure 4:**
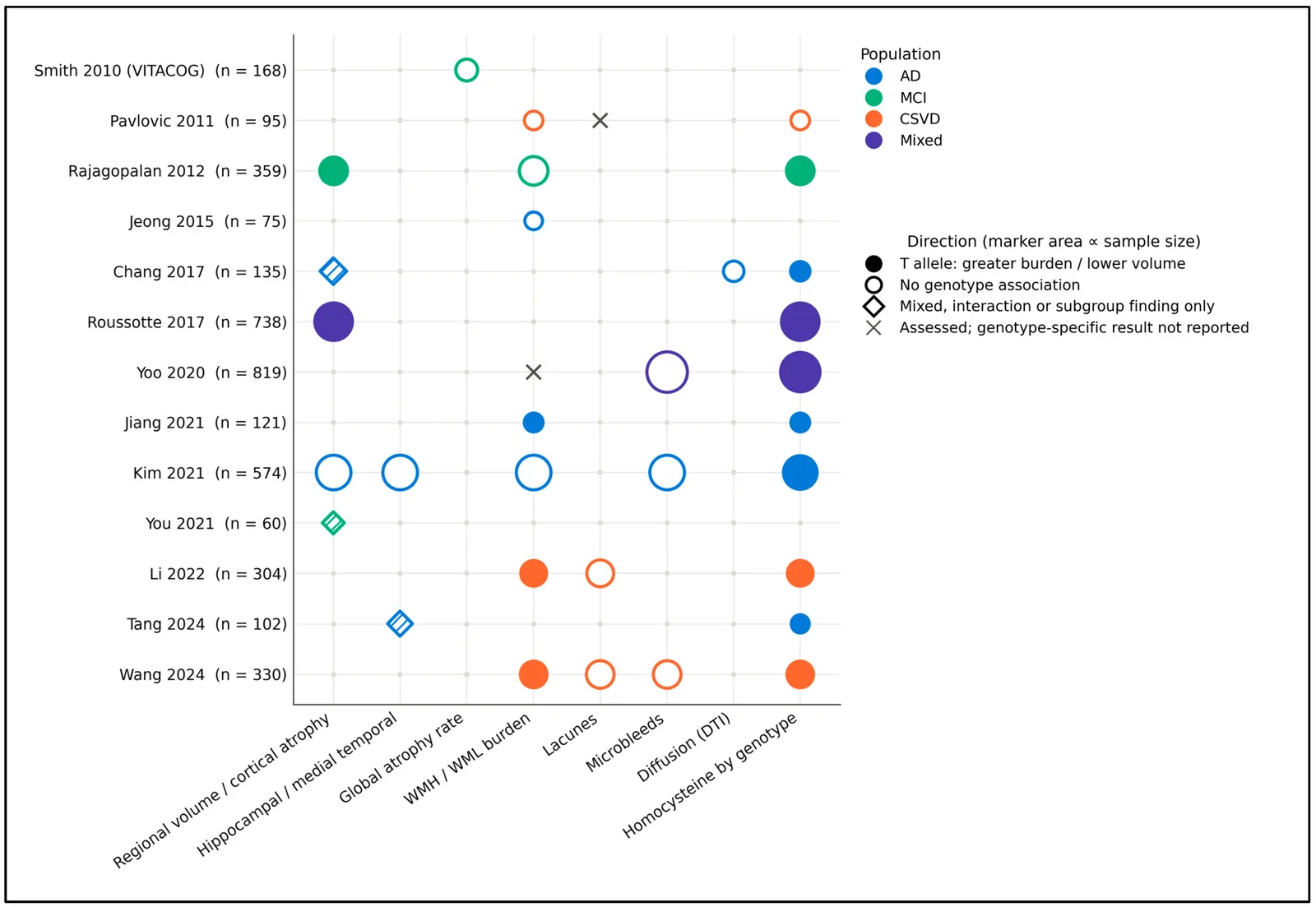
Evidence map of genotype–outcome comparisons in the 13 included studies. Filled markers denote a reported association of the T allele with greater lesion burden, lower volume, or higher homocysteine; open markers denote no genotype association; diamonds denote findings limited to an interaction or subgroup; crosses denote outcomes that were assessed but not reported by genotype. Marker area is proportional to the sample with genotype and MRI data; colour denotes the clinical population. AD, Alzheimer’s disease; CSVD, cerebral small vessel disease; DTI, diffusion tensor imaging; MCI, mild cognitive impairment; WMHs, white matter hyperintensities; WMLs, white matter lesions.

Taken across imaging domains, the pattern of findings is itself the main result. The only outcome for which several independent studies report concordant genotype differences is white matter lesion burden, where TT homozygotes carry a modest excess that is visible in unadjusted counts and in two adjusted models and is attenuated or absent in heterozygotes. Regional volumetric differences were reported in the two overlapping ADNI analyses and, as an isolated seed-level finding, in one AD cohort, but were not reproduced by the independent volumetric studies. Every comparison involving hippocampal or medial temporal structures, whole-brain atrophy rate, lacunes, microbleeds, or diffusion metrics was null, or positive only within an interaction or subgroup, while the genotype–homocysteine gradient was present wherever it was measured. The structural signal in this literature is therefore narrow rather than diffuse: it is concentrated in a vascular outcome, graded by genotype only in homozygotes, and derived from studies of predominantly moderate methodological quality with small TT subgroups.

**Table 4:** Methodological quality of the included studies on the adapted Newcastle–Ottawa Scale.

Study [Ref]	Selection (0–4)	Comparability (0–2)	Outcome (0–3)	Total (0–9)	Concern	TT Homozygotes (n)	Principal Limitations
Smith 2010 (VITACOG) [20]	3	1	2	6	Moderate	17	17 TT completers; genotype-specific confidence intervals internally inconsistent in supplement
Pavlovic 2011 [50]	3	2	1	6	Moderate	17	17 TT; recessive contrast only; adjusted OR coding unclear
Rajagopalan 2012 [51]	4	2	2	8	Low	53	Caucasian participants only; WMH result reported as *p* value only
Jeong 2015 [52]	3	0	2	5	Moderate	NR	No adjustment; CT/TT split not reported; genotype table inconsistent
Chang 2017 [53]	2	2	2	6	Moderate	6	6 TT; covariance interactions uncorrected
Roussotte 2017 [54]	4	2	3	9	Low	104	Overlaps Rajagopalan et al.; cross-sectional mediation
Yoo 2020 [55]	3	0	1	4	Moderate	141	Diagnostic subtype not reported; genotype absent from multivariable model; CC/CT counts not reported
Jiang 2021 [56]	2	0	1	3	High	10	10 TT; sub-cohort selection and rater blinding not described; unadjusted
Kim 2021 [57]	4	2	1	7	Low	NR	Genotype-specific imaging statistics not reported; many uncorrected comparisons
You 2021 [58]	2	2	2	6	Moderate	12	12 TT; interaction uncorrected; no genotype effect sizes
Li 2022 [59]	2	2	2	6	Moderate	59	Cognition not quantified; genotype coding unspecified; table percentages inconsistent
Tang 2024 [60]	2	2	2	6	Moderate	NR	CT/TT split not reported; stratum effect exploratory; overlaps You et al.
Wang 2024 [61]	3	2	3	8	Low	69	Retrospective single-centre; homocysteine as medians only

Selection items: defined or consecutive sampling frame; ≥100 genotyped and scanned participants with ≥20 TT homozygotes; validated genotyping with quality control; established diagnostic criteria. Comparability items: age and sex accounted for; additional confounders accounted for. Outcome items: blinded or automated imaging assessment; multiple comparison control or prespecified primary contrast with stated genetic model; complete genotype-specific reporting with effect sizes and uncertainty. Concern: low (7–9), moderate (4–6), and high (0–3). NR, not reported.

## 4. Discussion

This systematic review of 13 imaging genetics studies shows that the MTHFR C677T polymorphism has a consistent biochemical footprint but an inconsistent structural one in people with cognitive disorders. Wherever homocysteine was measured, T-allele carriers and especially TT homozygotes had higher concentrations, with a pooled excess of about 3 µmol/L for carriers and 5 µmol/L for homozygotes. Regional volume deficits associated with the T allele were reported only in the two overlapping ADNI analyses and, as a single-seed finding, in a Taiwanese AD cohort [51,53,54], whereas the whole-brain atrophy rate in VITACOG, hippocampal subfield and medial temporal measures, diffusion metrics, lacunes, and microbleeds showed no genotype-only association in any study [20,53,55,57,59,60,61]. White matter lesion burden is the only domain with a reproducible signal: TT homozygotes had roughly 1.7-fold higher unadjusted odds of moderate-to-severe lesions with no between-study heterogeneity, but the effect was not evident in heterozygotes in two of four cohorts, and the two AD studies with dominant model data pointed in opposite directions [52,56]. That the genotype–homocysteine gradient was present even in the studies whose imaging findings were null suggests a weak, conditional, or domain-specific link between circulating homocysteine and detectable structural change.

Regional and whole-brain findings should be distinguished. A variant might associate with local tissue differences without a detectable effect on global atrophy, particularly when a regional analysis searches many locations and a global analysis averages them. Rajagopalan et al. provide the only longitudinal regional evidence, and their 12-month white matter finding is consistent with a vascular rather than a neurodegenerative locus of effect [51]; Roussotte et al. extend the cross-sectional map to cortical regions in a diagnosis-adjusted cohort that shares participants with the first report [54]. A non-significant result in a small group of TT homozygotes does not establish equivalence between genotypes, and this applies to the VITACOG subgroup, to the Taiwanese diffusion comparison, and to the Korean WMH comparison with 75 participants [20,52,53]. Nevertheless, the largest AD cohort reported no association with several visually assessed structural outcomes [57], and the hippocampal subfield study found no genotype-only difference after correction [60]. Taken together, these observations constrain a general claim that C677T worsens the structural manifestations of AD.

The positive lesion findings in Chinese AD and CSVD cohorts are compatible with a vascular contribution, and the pooled recessive estimate summarises them, but they do not identify a common adjusted effect size. Differences in grading systems, covariate adjustment, recruitment, and lesion definitions [56,59,61] prevent these estimates from being read as successive confirmations of an identical effect. The recessive pattern, in which heterozygotes resemble CC homozygotes, is biologically plausible because enzyme activity in CT heterozygotes is only modestly reduced and their homocysteine excess is small.

The distinction between symptomatic CSVD and established vascular cognitive impairment is important. Results from the symptomatic CSVD cohorts, in one of which cognition was not quantified [59], should not be relabelled as findings in clinically diagnosed VaD. No included study provides a robust direct comparison of genotype effects between biomarker-defined AD and a well-characterised VaD group, so the available evidence cannot establish preferential involvement in either disorder.

Earlier meta-analyses addressed susceptibility to AD, VaD, MCI, CSVD, or lacunar infarction [14,15,16,17,18,19,25,26]. Those questions differ from whether genotype relates to structural burden within an already affected population. It is notable that the modest white matter effect observed here in TT homozygotes is of the same order as the pooled susceptibility odds ratios for VaD and CSVD reported in East Asian populations [16,25].

Studies outside the eligibility criteria provide useful context. The Rotterdam report found no C677T association with white matter lesions in non-demented participants [62], the geriatric depression report described an age-dependent WMH result [63], and hospital-based series without cognitive characterisation have reported both null and positive genotype associations [44,45]. None should be pooled with cognitively impaired cohorts, but they argue against assuming an association found in a selected clinical cohort will generalise across older adults. The homocysteine literature remains biologically relevant but does not resolve the genotype question: homocysteine integrates nutritional status, renal function, medication use, and age as well as genotype [9]. The variant and the circulating biomarker are related exposures rather than substitutes.

Folate and vitamin status are credible effect modifiers because they influence the biochemical expression of reduced MTHFR activity [8,9]. Tang et al.’s combined low-folate/T-carrier finding is consistent with this possibility, but the absence of a significant difference across all three risk strata after correction limits how strongly the subgroup pattern can be interpreted [60]. Between-country comparisons cannot isolate folate exposure because recruitment period, food fortification and supplementation, renal function, vascular burden, ancestry, diagnostic practice, and MRI methods differ simultaneously. The concentration of positive white matter findings in Chinese cohorts, where genotype-specific homocysteine differences were also largest, is therefore a hypothesis about gene–nutrient interaction rather than a demonstration of it.

The attenuation of the ADNI association after adjustment for homocysteine and the reported mediation estimate provide mechanistic clues [51,54], but they do not establish that homocysteine causally transmits the genotype effect. Cross-sectional mediation depends on temporal ordering and on assumptions about mediator–outcome confounding that these data cannot verify. VITACOG demonstrated a treatment-associated reduction in brain atrophy and an interaction with baseline homocysteine, while its MTHFR genotype-by-treatment interaction was not significant [20]. Randomisation of vitamin treatment strengthens causal inference about that intervention, but it does not randomise genotype or identify the biochemical component responsible for benefit.

The reviewed evidence does not support routine MTHFR testing to explain an individual patient’s MRI abnormalities, to distinguish AD from VaD, or to select vitamin therapy. None of the reports establishes the incremental value of genotype beyond age, vascular risk, cognitive assessment, standard imaging, and measured homocysteine or folate status, and a statistical association is different from a clinically useful prediction tool.

This review has several strengths. The eligibility criteria were specified before the searches were run and were restricted to genotype-stratified structural imaging contrasts in clinically defined cognitive populations. Genotype main effects were distinguished from interactions, mediation analyses, subgroup findings, and longitudinal results, so preliminary signals are not presented as replications. Crude effect estimates were recalculated from the published counts wherever possible, which exposed several internal inconsistencies in the source tables that are documented in the tables.

The evidence base is limited by heterogeneous diagnoses, small genotype subgroups, predominantly cross-sectional imaging, exploratory regional analyses, and incomplete reporting of uncertainty or null results. Differences in scanner field strength, segmentation, lesion scales, genetic models, covariate sets, and correction for multiple testing constrain comparability, and the pooled estimates rest on three to six studies and mix visual lesion scales with different grading rules. Three constraints govern how far the white matter finding can be taken. First, no included study adjusted the genotype–imaging contrast for the full set of age, hypertension and other vascular risk factors, renal function, folate and vitamin B12 status, and ancestry, and the pooled estimates are unadjusted throughout, so the observed association may reflect confounding rather than an independent effect of C677T. Confounding by ancestry is of particular concern because dietary folate intake, vascular risk profile, and other population characteristics may differ systematically between the East Asian and European cohorts that contribute to the estimate, and the positive findings are concentrated in the former. Second, statistical power for genotype contrasts is low: fewer than 20 TT homozygotes were available in five studies, and the TT count was not reported in a further three (Table 4), which makes recessive model estimates and interaction analyses involving folate status, APOE genotype, or treatment allocation unstable. Third, several of the influential studies are more than a decade old and used 1.0 to 1.5 T acquisitions, visual lesion scales, and clinical rather than biomarker-based diagnostic criteria, so they do not reflect current imaging or diagnostic standards [5,24].

Embase, Web of Science, Scopus, and Chinese-language databases were not searched, so further eligible reports may exist. Because most positive MTHFR findings have arisen in East Asian populations, the omission of Chinese-language databases is the most consequential gap in coverage. The rule-based triage could have excluded eligible reports whose terminology was not recognised, and screening and extraction were carried out by a single reviewer rather than by two independent reviewers. The absence of a prospectively registered protocol makes departures from the original analysis plan difficult to establish, and with fewer than ten studies per analysis, small-study effects and publication bias could not be assessed reliably.

Six methodological priorities follow directly from these constraints. First, longitudinal multimodal MRI, because cross-sectional data cannot distinguish differences that precede cognitive decline from those that accompany it or follow selection into a symptomatic cohort. Second, biomarker-defined AD cohorts, because clinically diagnosed samples contain mixed pathology that can both generate and conceal apparent genotype effects [1,23,24]. Third, standardised imaging pipelines, with quantitative WMH volume in place of visual grades, harmonised acquisition and processing, and reporting consistent with current small vessel disease standards [4,5]. Fourth, adequately powered genotype groups, sized for the recessive model and for the gene–nutrient interactions of interest, with documented genotyping quality control and ancestry adjustment. Fifth, complete genotype-specific reporting: counts, effect estimates, and measures of uncertainty for null as well as positive contrasts, with the reference genotype and the coefficient coding stated. Sixth, individual participant data synthesis, which would permit confounder-adjusted pooling, formal testing of effect modifiers, resolution of cohort overlap, and estimation of the incremental value of genotype over homocysteine, nutritional status, vascular risk, and conventional imaging measures [64].

## 5. Conclusions

In people with Alzheimer’s disease, mild cognitive impairment, and vascular cognitive disorders, the MTHFR C677T polymorphism is reliably associated with higher plasma homocysteine but only inconsistently with structural brain abnormalities. The principal contribution of this synthesis is to show that the inconsistency is not random but patterned by outcome domain: the available evidence is more compatible with a modest association with vascular white matter injury, concentrated in TT homozygotes, than with a reproducible neurodegenerative imaging phenotype. All three positive white matter lesion findings came from Chinese cohorts, whereas the Korean, Serbian, and US cohorts that reported genotype-specific white matter lesion results showed no association; whether this pattern reflects a gene–nutrient interaction related to folate status, ancestry, or other between-population differences remains a hypothesis that the present data cannot test. Regional volume associations were largely confined to two overlapping analyses of a single cohort, and hippocampal, medial temporal, global atrophy, lacune, microbleed, and diffusion measures showed no genotype-only association in any study. The practical implication is explicit: current evidence does not support genotype-guided clinical decision-making, whether to explain an individual patient’s imaging findings, to distinguish Alzheimer-type from vascular contributions, or to select vitamin therapy. Establishing clinical relevance would require longitudinal multimodal imaging in biomarker-characterised cohorts, harmonised quantitative lesion measurement, genotype groups large enough to support recessive and gene–nutrient interaction models, complete genotype-specific reporting, and individual participant data analyses capable of separating the contribution of genotype from those of homocysteine, nutritional status, vascular risk, and ancestry.

## Figures and Tables

**Figure 1:**
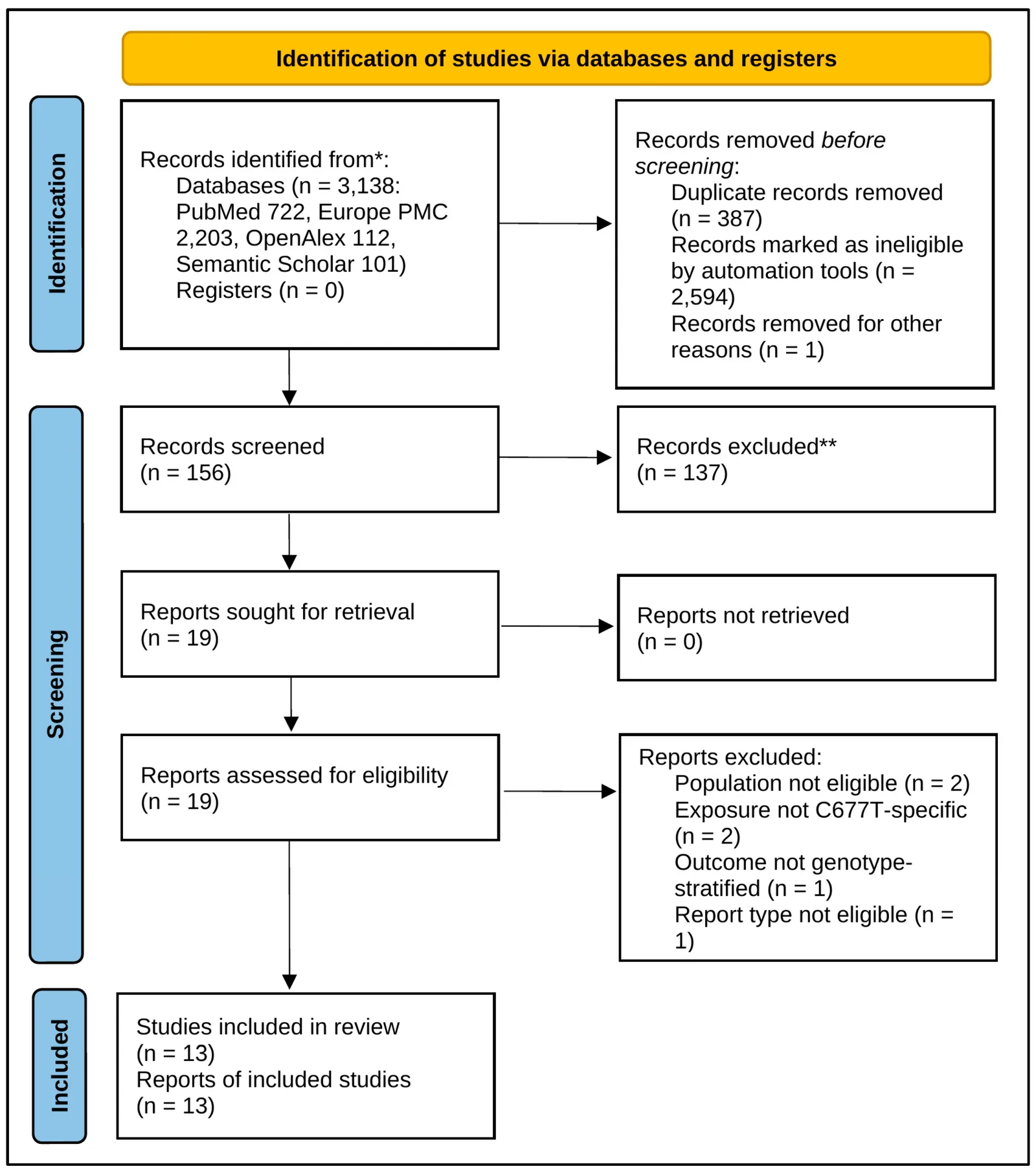
PRISMA 2020 flow diagram of study selection [27]. * Records identified per database: PubMed 722, Europe PMC 2203, OpenAlex 112, and Semantic Scholar 101. ** The 137 exclusions at screening were made by the reviewer; the 2594 records marked ineligible before screening were removed by rule-based automation (publication type, animal-only, absence of an imaging, cognitive, or MTHFR concept), and the titles of these records were additionally inspected by the reviewer. Forward citation searching (959 records) yielded no additional eligible reports.

## Data Availability

The search strategies, record-level screening ledger, extracted data, and analysis code are available from the corresponding author upon reasonable request. No new primary data were created in this study.
